# Islands as Hotspots for Emerging Mosquito-Borne Viruses: A One-Health Perspective

**DOI:** 10.3390/v11010011

**Published:** 2018-12-25

**Authors:** Carla Mavian, Melissa Dulcey, Olga Munoz, Marco Salemi, Amy Y. Vittor, Ilaria Capua

**Affiliations:** 1 Department of Pathology, Immunology and Laboratory Medicine, College of Medicine, University of Florida, Gainesville, FL 32611, USA; salemi@pathology.ufl.edu; 2 Emerging Pathogens Institute University of Florida, Gainesville, FL 32611, USA; dulceym@ufl.edu (M.D.); omunoz@ufl.edu (O.M.); Amy.Vittor@medicine.ufl.edu (A.Y.V.); icapua@ufl.edu (I.C.); 3 Department of Environmental and Global Health, College of Public Health and Health Professions, University of Florida, Gainesville, FL 32611, USA; 4 One Health Center of Excellence, University of Florida, Gainesville, FL 32611, USA; 5 Division of Infectious Diseases and Global Medicine, Department of Medicine, College of Medicine, University of Florida, Gainesville, FL 32611, USA

**Keywords:** One-Health, island, hotspot, Indian, Pacific, Caribbean, arbovirus, dengue, zika, chikungunya

## Abstract

During the past ten years, an increasing number of arbovirus outbreaks have affected tropical islands worldwide. We examined the available literature in peer-reviewed journals, from the second half of the 20th century until 2018, with the aim of gathering an overall picture of the emergence of arboviruses in these islands. In addition, we included information on environmental and social drivers specific to island setting that can facilitate the emergence of outbreaks. Within the context of the One Health approach, our review highlights how the emergence of arboviruses in tropical islands is linked to the complex interplay between their unique ecological settings and to the recent changes in local and global sociodemographic patterns. We also advocate for greater coordination between stakeholders in developing novel prevention and mitigation approaches for an intractable problem.

## 1. Introduction

The term “arbovirus” (arthropod-borne virus) describes viruses transmitted by blood-feeding arthropods [1]. Most arboviruses are maintained in complex sylvatic cycles involving forest-dwelling mosquitoes and vertebrates that serve as reservoirs or amplification hosts, such as birds, small mammals and non-human primates [1]. Spillover from these sylvatic cycles occurs occasionally to cause disease in humans, and subsequent viral adaptation can then give rise to urban or epidemic cycles [2,3].

In our review, we identify drivers of arboviral emergence specific to island settings. As such, we focus on the two most important arboviral genera in our regions of interest (genus *Alphavirus*, family *Togaviridae* and genus *Flavivirus*, family *Flaviviridae*), transmitted by *Aedes* spp. and *Culex* spp. mosquitoes.

Over the last decade, an increasing number of arboviral outbreaks has been registered, rapidly spreading throughout the tropics and subtropics upon importation into these areas from endemic regions of Africa and South-East Asia [4,5,6]. In 2005, outbreaks of chikungunya virus (CHIKV) were registered in the Indian Ocean islands [7], and infections were associated with not only a febrile illness with protracted joint pain, but also neurological complications, fetal infections and a rise in overall mortality rates [8,9,10]. This viral strain of East, Central and South African (ECSA) lineage was later discovered to have undergone a key point mutation leading to adaptation of the virus to *Aedes albopictus* [11], a mosquito vector that had previously become established on many Indian Ocean islands. This outbreak spread to India and South-East Asia in 2006, causing explosive outbreaks [12]. In 2011, a different CHIKV strain (of Asian lineage) was first detected in the Pacific Ocean, on the island of New Caledonia [12]. Following its spread to French Polynesia, CHIKV was detected in 2013 in Saint Martin, marking the first recorded autochthonous transmission of CHIKV in the Americas [13]. Shortly thereafter, CHIKV was reported in more than 40 other countries in the Caribbean and the Americas [7].

Zika virus (ZIKV) probably emerged centuries ago from Africa into South-East Asia [14]. Similar to the second wave of CHIKV outbreaks, the recent ZIKV pandemic ensued following the spread of an Asian ZIKV strain into the Oceania and then the Americas [14]. Prior to its appearance in the Pacific and the Americas, Zika virus (ZIKV) infections were sporadic and caused only mild symptoms [15,16]. However, well into the epidemic in Brazil in 2015, it became apparent that ZIKV infection could lead to microcephaly, other congenital malformations, and Guillain-Barré syndrome. By 2016, ZIKV was widely distributed in the Americas and the Caribbean islands constituting yet another arboviral public health concern [17]. The dispersal pattern of the recent explosive epidemic waves of CHIKV and ZIKV [13,18], as well as earlier waves of dengue outbreaks [19], have drawn attention to islands as potential hubs for arboviral emergence, facilitating dispersal to the mainland and fueling of global epidemics [5,6,20,21]. Recent altered ecologic conditions, due to changes in global climate (increasing temperatures and rainfall) and human behavior (global commerce and movement, urbanization) already represent drivers of expansion for arboviruses. Increased vector density and distribution, due to vector adaptation to residential environment and unsuccessful or unsustainable vector control programs, have intensified the potential of new arboviruses to emerge from enzootic cycles and spread to new geographic areas with establishment of new endemic cycles both in humans and animal reservoirs [4,21]. These drivers may be exacerbated in islands by unique or peculiar circumstances present on these small landmasses. Tropical oceanic islands have a variety of commonalities related to limited access to public health resources, and intense movement of human and goods along commercial and cruise ship routes. Such islands host potential vectors for arthropod-borne viruses to which autochthonous populations may be naive. In addition, islands are particularly vulnerable to natural disasters (i.e., hurricanes, earthquakes, and volcanic eruptions) and their consequences can be completely disruptive, transformational and long lasting, further contributing to the environment’s capacity for arboviral transmission and maintenance [6,22]. These conditions have set the stage for the global dispersal of dengue (DENV), CHIKV, and recently ZIKV, and may promote novel, unknown and unexpected arboviral threats to public health.

In this review, we sought information on the role of Indian Ocean-, Pacific- and Caribbean islands as arboviral hotspots. We performed an extensive survey of past and current literature to gather information on emergence of arboviruses on islands, and investigated how the socioeconomic and climatic drivers affect insular host–vector–pathogen dynamics. Finally, we draw attention to possible complementary public health strategies that can be implemented in order to enhance control of unexpected arboviral emergence and spread.

## 2. Materials and Methods

We reviewed emergence of mosquito-borne viruses on islands belonging to three different geographic areas: the Indian Ocean, Pacific Ocean, and Caribbean. A full list of small islands nations and archipelagos is provided in Table 1. We searched from the 1960s to date (as of June 2018) for first reports (serology or viral detection in human, non-human or vector) of mosquito-borne arboviruses by island nation or archipelago. We searched for the string arbovir*, as well as individual arboviral terms (zika, chikungunya, dengue, Rift Valley fever, Ross River) followed by the name of the island or archipelago in PubMed and ProMED databases. The Pan American Health Organization (PAHO) and World Health Organization (WHO) webpages were utilized to achieve a more comprehensive understanding of virus distribution. The inclusion criterion for the articles and websites was any material published after and including 1960 and published in English, French or Spanish. It is important to take into account that the ProMED database was established only in 1994. As we reported only the first report of an arbovirus in an island, the literature search for that arbovirus-island pair ended once the first record was found. Different dengue serotypes are distinguished when possible, but recurrences of previously noted virus or dengue serotypes are not reflected in our search. Table 1 summarizes these first reports of specific arboviral presence based on human case reports, surveillance reports, serological studies in humans, as well as epidemiological studies in vectors and other animal hosts.

We collected climate, economic, and travel data searching in Pubmed and Google, using as strings for our search “arbovirus”, as well as individual arboviral terms (zika, chikungunya, dengue, Rift Valley fever, Ross River), followed by “island” and specific area (Pacific, Indian, Caribbean) and by: climate, climate change, socio-economics, sustainability, vector distribution, travel, or One-Health.

## 3. Results

### 3.1. Indian Ocean Islands

It is likely that waves of arboviral epidemics have occurred on these islands over centuries [23]. DENV (genus *Flavivirus*, family *Flaviviridae*), Rift Valley Fever (RFV; genus *Phenuiviridae*, family *Phlebovirus*), CHIKV (genus *Alphavirus*, family *Togaviridae*) have been detected in the Indian Ocean Islands, and serological studies provide further evidence for Sindbis virus (SINV), Kyasanur Forest disease (KFD; genus *Flavivirus*, family *Flaviviridae*), Japanese encephalitis (JEV; genus *Flavivirus*, family *Flaviviridae*), West Nile virus (WNV; genus *Flavivirus*, family *Flaviviridae*), Langat virus (LGTV; genus *Flavivirus*, family *Flaviviridae*) circulation (Table 1).

The first clinically diagnosed documentation of in the Indian Ocean Islands occurred during a DENV fever outbreak with 59 cases in Comoros in 1943 [24]. DENV serotype 2 (DENV-2) was reported for the first time in the Seychelles and Reunion in 1977 [25,26]. DENV was reported again in 2004-2005 [27], with PCR-confirmed DENV-1 and 3, co-circulating with CHIKV [23]. DENV-1 emerged in the Seychelles in 2016 [28]. Although there was no record of laboratory-confirmed cases in Mauritius during the 1977–1978 DENV-2 epidemic, an epidemiologic study conducted in 1994 suggested that DENV was circulating in the island [29,30]. DENV was first reported in the Maldives in 1979, and has caused sporadic outbreaks until it became endemic in 2004 [31]. In 2011, DENV-1 was found to responsible for the largest epidemic in Malé and other islands of the Maldives [32]. In the eastern Indian Ocean, a serological survey realized between 1988–1989 among the Andaman and Nicobar group of islands in India found evidence of DENV-2, together with high prevalence of the tick-borne KFD, as well as of other arboviruses such CHIKV, JEV, WNV, LGTV [33]. Since its first documentation on these islands [33], DENV was not reported again until 2009-2010 (co-circulation of DENV-1 and DENV-2) [34]. DENV-3 was then identified for the first time in 2013 in Port Blair, the capital of the Andaman and Nicobar Islands [35].

RVF appeared in the Indian Ocean in 2004 in livestock on the island of Mayotte [36]. Human cases of RVF were reported in 2007–2008, and again later in 2011 (Table 1). Possible introduction of RVF in the Comoros Islands from Africa occurred in August 2007 when autochthonous RVF transmission in humans was reported for the first time on the island of Grande Comore, with subsequent spread to other islands including Mayotte [37].

The earliest reports of CHIKV are from a dengue epidemic surveillance effort in 1977 in the Seychelles that led to the first evidence of CHIKV and Sindbis (SINV) in the Indian Ocean [38]. Human sera were found to be positive for monotypic neutralizing antibodies to CHIKV (8% of 231 patients sampled) and SINV (7% of 231 patients sampled) [38]. Another serological survey in 1988 found presence of CHIKV in the Andaman and Nicobar islands [33]. Circulation of CHIKV went mostly unnoticed in the Indian Ocean until 2005, when CHIKV caused the explosive outbreak in Reunion Island with 255,000 cases reported between March 2005 and April 2006 (Table 1) [39,40]. The outbreak then spread to Mayotte, Mauritius, the Seychelles, and Madagascar [40]. Phylogenetic analysis indicated that the circulating CHIKV constituted a new lineage (dubbed the Indian Ocean lineage, a descendant of the East, Central, South African (ECSA) clade) [40]. A hallmark of this epidemic was the A226V mutation that allowed for a higher replication and dissemination rate in the *Aedes albopictus* mosquito, resulting in enhanced transmission efficiency [11]. Whereas CHIKV was associated previously with the urban *Ae. aegypti* mosquito (possibly aided by *Ae. albopictus*) in Asian countries, the 2005 outbreak was mainly transmitted by *Ae. albopictus.* This increased the likelihood of CHIKV-infected travelers giving rise to autochthonous transmission upon their return home, since *Ae. albopictus* vectors are indigenous in many areas of Europe and the Americas [11,40].

### 3.2. Pacific Ocean Islands

Six years after the outbreak in Reunion, CHIKV emerged in the Pacific Ocean in New Caledonia in 2011 [20] (Table 1). Before this outbreak, local transmission of the virus had not been seen in the region [20]. Outbreaks followed in Melanesia in 2012 and Micronesia in 2013. By 2014, CHIKV was spreading through American Samoa, French Polynesia, Samoa, Tokelau, and Tonga [41].

DENV is the most widespread virus throughout the Pacific, with all four DENV serotypes found in the Pacific area (Table 1). Most islands have co-circulation of two serotypes (commonly DENV-1 with one of the other serotypes), but New Caledonia has reported co-circulation of all serotypes (Table 1). Major outbreaks of DENV-1 across the Pacific are believed to have commenced following an index outbreak in Palau in 2000 [42]. DENV in the Pacific was recorded in Hawaii in 2001 when autochthonous DENV-1 infections were linked to travelers returning from Tahiti, French Polynesia [43]. Phylogenetic analyses suggested association of the Hawaiian strains with contemporaneous Polynesian strains [43]. Due to the different feeding behavior of *Ae*. *albopictus* as compared to *Ae. aegypti*, the 2001–2002 DENV-1 outbreak in Hawaii was more limited in number of cases as compared to the one that occurred at the same time in the Society Islands, where *Ae. aegypti* was responsible [43]. Emergence of new endemic lineages of DENV 1-3 in Papua New Guinea has been reported [44] as well as re-emergence of DENV-2 in the Solomon Islands in 2016 [44].

Despite some evidence of Ross River virus (RRV; genus *Alphavirus*, family *Togaviridae*; first isolated in 1959 in Australia [45]) seroprevalence in the mid-1960s in the Solomon Islands, RRV was not appreciated in the Pacific Islands until the 1979–1980 epidemic involving half a million cases [46,47,48]. During this time, the literature mentions outbreaks occurring in American Samoa, the Cook Islands, Fiji, New Caledonia, as well as Wallis and Futuna (Table 1). Subsequently, a serological study in 2011–2013 in French Polynesia detected RRV IgG in a third of blood donors, suggesting that there had been silent circulation of RRV [49].

The most recent arbovirus to emerge in the Pacific islands was ZIKV (genus *Flavivirus*, family *Flaviviridae*). Between 1963 and 1983, ZIKV was present in equatorial Asia, including Indonesia and Malaysia [18]. In 2007, the first large ZIKV outbreak occurred in Yap State, Micronesia, infecting approximately 73% of Yap residents [50]. The high incidence recorded during the ZIKV outbreak in Yap Island suggests a lack of immunity in this population. Additional ZIKV outbreaks occurred in French Polynesia in 2013, shortly followed by New Caledonia, the Cook Islands, and Easter Island in 2014 and later Fiji, Vanuatu, the Solomon Islands, Samoa, American Samoa, Tonga and the Marshall Islands (2015–2016) (Table 1).

### 3.3. Caribbean Islands

According to historical accounts, yellow fever virus (YFV; genus *Flavivirus*, family *Flaviviridae*) was introduced into the New World from Africa through the Caribbean islands of Barbados and Guadeloupe in 1647 in association with the African slave trade [4], although the disease may have been in Haiti as early as 1495 [51]. The islands of Martinique and Guadeloupe in 1635 witnessed the first suspected dengue-like epidemic reported in the New World [52,53]. It was only later, during a large dengue epidemic in 1827–1828 that originated in the Virgin Islands, then spread to Cuba and Jamaica [54,55], that the disease was named Dunga [55]. DENV-2 was first isolated in 1953 in Trinidad and Tobago [56,57]. In 1947, the Pan American Health Organization (PAHO) initiated a campaign to eradicate *Ae. aegypti* [58]. Despite initial success, the *Ae. aegypti* eradication campaign eventually failed, leading to the reappearance of dengue serotypes in the Caribbean in the 1970s. DENV-3 spread through the Caribbean in the epidemic of 1963–1964 [59]. DENV-1 appeared in Jamaica in 1977 [60], and DENV-4 first reappeared in 1981, possibly originating from the Pacific Islands [56]. This year was also marked the first detection of a dengue hemorrhagic fever (DHF) case in the Americas, which occurred in Cuba [61] (Table 1).

In 1969 an outbreak of eastern equine encephalitis virus (EEEV; genus *Alphavirus*, family *Togaviridae*) was registered in Cuba [62], however it was only the 1977 virologic and serological survey of wild vertebrates that demonstrated that birds were the main hosts of EEEV in forest and water–litoral regions of various provinces of Cuba [63]. Venezuelan equine encephalitis virus (VEEV; genus *Alphavirus*, family *Togaviridae*) was isolated from Trinidad and Tobago between the 50s and 60s [64]. Around the same time, Mayaro virus (MAYV; genus *Alphavirus*, family *Togaviridae*) was first isolated in Trinidad (1954) [65]. It was not until 2014 that MAYV was recorded again in the Caribbean, in Haiti [66]. One year after its re-emergence, a new recombinant form of the virus was isolated in Haiti [67,68] (Table 1). In Trinidad, between 1955 and 1959 the viruses Oropuche virus (OROV), Bushbush virus (BSBV), Ieri virus (IERIV), Lukuni virus (LUKV), belonging to the genus *Peribunyaviridae*, family *Orthobunyavirus*, were found in mosquitoes [69,70], as well as St. Louis encephalitis virus (SLEV) was detected in birds and mosquitoes [71,72]. Low-level circulation of and Madariaga virus (formerly known as South American EEEV), together with VEEV, was found from samples collected from seropositive horses in Trinidad between 2006–2009 [73].

Three cases of Ilheus virus (ILHV) were registered in Trinidad in 1962, from a city resident with severe febrile illness, and two mosquito catchers working in the forests of the island of which only one reported mild febrile illness [74]. Restan virus (RESV) was also found in Trinidad in 1965 in mosquitoes [75]. Mucambo virus (MUCV) was found in mosquitoes and mammals in 2007 [64].

The first human WNV case in the Caribbean was detected in 2001 in the Cayman Islands, probably having reached the island via migratory birds [76]. Until now, only nine additional human cases have been detected in the region (Table 1). In 2004 WNV was reported in horses and birds in Trinidad [77].

CHIKV was first detected in Saint Martin in December 2013 [78], and although this is considered the first case of CHIKV in the Americas [79], it is possible that a previous outbreak had occurred (Table 1). Historical records of the 1827–1828 DENV epidemic suggest that this epidemic might have been erroneously attributed to DENV, as the clinical characteristics were more similar to disease seen with CHIKV infections. [80]. Between the end of 2013 and throughout 2014, the virus was detected in virtually every Caribbean country. Interestingly, phylogenetic analysis indicates that the strain responsible for the CHIKV outbreak in French Polynesia in 2014 originated from a traveler returning from Guadeloupe in the Caribbean [41]. These analyses have also demonstrated that the Indian Ocean CHIKV outbreaks were due to CHIKV of the East/Central/South African (ECSA) lineage, while the Pacific Island and Caribbean epidemics were attributed to CHIKV belonging to the Asian lineage [81].

The most recent arbovirus to have emerged in the region is ZIKV, with the first case detected in Puerto Rico in a man with onset of symptoms beginning on November 23, 2015. Between the end of 2015 and throughout 2016, similarly to CHIKV, ZIKV spread in less than one year to every Caribbean country (Table 1). The establishment of autochthonous infections in the Caribbean fueled further exportation of the virus to Europe [82] and the US [83].

### 3.4. One-Health Perspective

The One-Health perspective integrates social, economic, biomedical and ecological fields of study with the community of policymakers and practitioners working on the ground to have the most impact. In this section, we focused on One-Health factors that have contributed to the persistence and spread of arboviral outbreaks in the context of insular tropical environments. We particularly investigated socio-economic determinants, climatic conditions, human migration and trade as potential drivers for arboviral emergence in islands, and their impact on host-vector-pathogen dynamics.

#### 3.4.1. Socio-Economic Drivers

Poverty is one of the main drivers for arboviral infections [84]. Indeed, ZIKV [85], DENV [86] and CHIKV [87] outbreaks have been linked to populations with vulnerable socioeconomic status. Poverty may lead to inadequate vector control and prevention programs, suboptimal healthcare infrastructure, and the creation of environmental conditions that promote mosquito breeding [84].

In 1992, the United Nations recognized Small Island Developing States (SIDS) as a specific group of developing countries [88,89]. At present, 57 countries from the Caribbean, the Pacific, the African and Indian Ocean, the Mediterranean, and South China Sea, have been designated as such [88]. SIDS are joined by similar opportunities but especially by challenges for attaining sustainable development. These include limited resources, remoteness, a great reliance on international trade, fragile environments, small but growing populations, transportation and energy costs, and difficulties in creating economies of scale [90,91,92].

Another layer of complexity comes from the differences in sovereignty between the various islands and archipelagos. For example, within the Caribbean, seventeen islands are territories of more economically developed countries (with differing status within the sovereign country), and 13 are independent states. These differences can influence the type and quantity of help and attention they receive, as well as the coordination of efforts in these island states.

Regarding economic challenges, Caribbean independent states have expressed the willingness to integrate into the global market. Nonetheless, given their small size and less developed economic and export structure, it is recognized that they might need special treatment [93].

SIDS face further challenges in financing public administration and infrastructure, due to the disproportionate expenses these entail given the small size of their economies [90]. In addition to these obstacles, SIDS also encounter the many burdens that other developing countries experience, such as disorganized urbanization coupled with non- or under-developed water distribution systems and waste management [94]. The absence or deficiency of water and waste systems is associated with a rise in the use of water storage containers, which in turn facilitate the proliferation of *Aedes* mosquitoes [94]. Further socio-economic factors related to poverty that affect the distribution of *Aedes sp.*, are air-conditioning and housing quality [95]. 

#### 3.4.2. Climatic Conditions and Climate Change

Climate has a profound impact on the spatiotemporal distribution of arthropods, the vector life cycle, arboviral dispersal patterns and evolution, and transmission efficiency from vector to host [96].

Oceanic regions are disproportionately affected by anthropogenic climate change and extreme weather events (e.g., hurricanes, cyclones and tsunamis) [97]. Areas of low elevation and coastal areas will experience some of the greatest consequences of severe weather events and sea level rise [98,99,100]. Natural catastrophes including hurricanes, cyclones and storm surge can wipe-out or severely alter areas of vegetation, and human dwellings on islands [100]. Sea level rise may result in the loss of large portions of land or complete submergence of islands. These elements have the ability to greatly impact the landscape and biodiversity of islands [101], which could also increase vector density. Due to their low elevation, islands such as the Maldives [102], the Marshall Islands [103], and Tuvalu [104,105] are currently considering resettlement as they deal with the effects of climate change, including sea level rise, high tides and frequent storms. These situations result in the movement of people and animals, resulting in opportunities for increased human, host and vector exposure and interactions.

Some factors believed to be impacted by climate change and linked to alterations in vector-borne diseases include but are not limited to temperature, precipitation, flooding, daylight, wind, and humidity [106]. Naturally, these types of changes would alter the host and vector population dynamics and ecology, which could lead to disease emergence. *Ae. aegypti and Ae*. *albopictus* exhibit great ecological plasticity and potential for spread to new settings, allowing these mosquitoes to adapt to new environmental conditions, feeding behaviors, and breeding sites [107,108]. For example, extraordinary high temperatures recorded at *Ae. aegypti* breeding sites in Trinidad caused water evaporation and mortality of immature stages, leading the mosquito to establish new breeding sites in underground drains and septic tanks without affecting the fitness of *the vector* [109]. Increased availability of brackish water due to climate change and natural disasters could increase available breeding sites for vectors that are able to proliferate in salt-water pools. Larval development of *Ae. aegypti* and *Ae. albopictus* in peri-urban brackish water was observed in Sri Lanka [110], and in the Andaman Islands, India in 2004, following a tsunami which resulted in increased breeding sites for *Anopheles sundaicus* [111]. These examples stress the importance of expanding existing guidelines on dengue control [112] to include brackish water habitats and underground breeding sites of *Ae. aegypti* and *Ae. albopictus*.

Changes in rainfall and extreme weather conditions are not specific to islands alone. However, smaller landmasses (specifically small islands) can be extremely susceptible to changes in precipitation due to a lack of infrastructure to manage changes in water flux [113]. This can give rise to open water sources for mosquitoes to proliferate. On the other hand, islands that experience drought and do not have infrastructure for water storage may rely on household water storage containers, thereby also increasing mosquito-breeding sites [94].

Severe storms with high winds such as hurricanes also have the ability to displace and move birds far outside their normal ranges [114]. As islands tend to have limited extension of land areas, birds that are caught and survive through these winds are likely to end up on other lands masses, taking with them the pathogens they carry and the possible introduction of new diseases. For example, the long-distance movement of birds, including their displacement through tropical storms, has been discussed as a possible mechanism for the spread of WNV to the Western Hemisphere [115]. Mosquitoes may also experience a similar phenomenon. Although it is possible for strong winds to decrease activity of mosquitoes, it also has the ability to move and disseminate vectors and the diseases they carry [116]. This is especially true for more closely located landmasses and is exemplified by the possible introduction of Japanese encephalitis from Papua New Guinea to Australia, by way of islands through the Torres Strait [117].

Recent history emphasizes how hurricanes are a prime example of how islands are especially vulnerable to natural disasters. When hurricane Maria made landfall in Puerto Rico in September of 2017, the island was left with around $90bn worth of damage, and households went without electricity for an average of 84 days and without water for an average of 64 days [118].

#### 3.4.3. Man-Related Activities Influencing Vector Dynamics

Man-related activities are probably one of the most important drivers that can act as amplifiers of infection. Over the centuries, geographic distribution of arboviruses and their vectors has been shaped by mobility of people and goods. Due to the progressive desertification of the Sahara Desert 4000–6000 years ago, human migration initiated the process of domestication of *Ae. aegypti* [119,120], thus increasing the burden of arboviral diseases. Another example where human movement played an important role in the emergence and movement of an arbovirus was the re-location of around 50,000 Haitians to the US [121] and migration of 4000 to 6000 Haitians across the Amazon [122] after the 2010 earthquake in Haiti. It has been proposed that this extraordinary human migration elicited the emergence of a new recombinant genotype of MAYV in Brazil, later exported to Haiti [68]. Ecological changes due to human land use is often implicated in sylvatic spillover events into the human population: deforestation due to encroachment of human settlements and agriculture increases human exposure to sylvatic vectors, leading eventually to the adaptation of the virus–mosquito–human cycle with further spread to peri-urban and urban settings [123]. Increasing urbanization is one of the main drivers for the expansion of *Ae*. *aegypti*, which is closely associated with human residential settings. Although *Ae. albopictus* was previously considered a rural or peri-urban vector [124,125,126], recent evidence of *Ae. albopictus* adaptation to urban life was found in urban settings in Guangzhou, China [127], and La Reunion where the vector was breeding in parks, residential gardens, and cemeteries [128].

Human trade has been an additional driver for displacement of vectors and pathogens [129]. During the 17th century, due to the implementation of freshwater storage systems in ships for long-distance travel [119] and the intense slave trade from Africa to the Caribbean after the establishment of the “Compagnie du Sénégal” [130], *Ae. aegypti* was exported from Africa to the French Antilles archipelago [120]. In the 1980s, maritime international trade of used tire shipments were important in the re-introductions of both *Ae. aegypti* in the Americas, but also for the dispersal of *Ae. albopictus* [131,132]. Dispersal of *Ae. albopictus* into the Pacific and Indian Ocean Islands started around the 17-18th centuries due to increased trade with Asia [133,134], and between 1800–1850, whaling and sandalwood commerce with China [135] led to first introductions of *Ae*. *aegypti* in New Caledonia, Fiji and Tonga [134]. *Ae*. *aegypti*’s infestation of the Pacific is an ongoing issue, as shown by the more recent colonization of the Isle of Pines (New Caledonia, Melanesia) in 2003 [134]. A recent study on seaports in the Philippines showed that cargo ship transportation is the main mode of dispersion of *Ae*. *aegypti*, and that movement of *Ae*. *aegypti* between cities is faster in highly-populated cities with large ports [136]. This study suggests that modern vector control techniques such as *Wolbachia* release may not be successful in such seaports due to continuous importation of new vector populations. Interestingly, the lack of *Ae*. *aegypti* in the Indian Ocean has protected this region from the ZIKV pandemic, as *Ae. albopictus* is not an efficient vector for the Asian ZIKV genotype [137].

The recent global spread of ZIKV [138,139] and CHIKV [140,141,142] highlighted the importance of a relatively new element that contributes significantly to epidemic spread, namely tourism. Modern international civil aviation allowed for a tenfold increase in global air travel over the last 40 years, drastically reshaping human interconnection [143] and facilitating spread of ZIKV in South, Central, and North America [85]. A recent study found a strong correlation between Caribbean cruise ship tourism and establishment of ZIKV in Florida. It is estimated that 2.4 million travelers arrived from the Caribbean into Miami via cruise ships between January and June 2016, accounting for around of 40% of the total traffic into Miami. The authors further estimate that during that period 60–70% of ZIKV-infected travelers had arrived to Florida from the Caribbean [83]. 

### 3.5. The Caribbean Hotspot

Due to their proximity, trade, and political relationships with the continental U.S., the Caribbean Islands facilitate arboviral spread into the U.S. At least six out of thirty Caribbean countries, such Bahamas, Haiti, Jamaica, Dominican Republic, Saint Kitts and Nevis, and Trinidad and Tobago, export equal to or greater than 40% of their products to the U.S. [144].

Florida, which includes the most southern tip of the United States, can act as a portal of entry for pathogens and invasive species into the U.S. for several reasons. Florida is geographically part of the Caribbean hotspot, includes large metropolitan areas, such Orlando and Miami, that represent major tourist destinations, and the tropical climate in the central and southern parts of the State is similar to the climate in the Caribbean islands. Both areas allow for proliferation of *Ae. aegypti* and *Ae. albopictus*. In addition to these, mosquito-borne viruses that have been found in Florida in the past decade include WNV, SLEV, EEEV, Everglades virus [145], Highlands J virus [146], and LaCrosse encephalitis virus (LACV) (Table S1).

In Florida, imported arboviruses tend to proliferate and generate autochthonous cases. WNV activity has been reported in humans as well as in horses and sentinel chicken from the Florida Panhandle to the Atlantic coast (Table S1). All four serotypes of dengue have been detected in the state and local transmissions have been reported in recent years (Table S1 and S2). [147]. The Florida Department of Health reported a total of 965 travel-associated ZIKV cases in 2016 [148], 195 cases in 2017 [149], and 66 cases through October 2018 [147]. Approximately 50–80% of the imported cases of DENV, CHIKV and ZIKV found in Florida through 2008–2018 were introduced from the Caribbean islands, mainly Puerto Rico, Dominican Republic, Haiti, Cuba and Jamaica (Table S2).

This has urged the state of Florida to react over the last decade with mosquito-borne illness alerts and advisories being routinely in place for certain counties with high prevalence or risk of these pathogens, such as Orange, Miami-Dade and Collier counties, where large CHIKV, DENV and ZIKV outbreaks have occurred [147].

## 4. Discussion

### 4.1. Potential Emerging Arboviral Threats

In 2017, the opinion piece “Could the recent Zika epidemic have been predicted?” was published [150] in response to the explosive Zika epidemic rapidly burdening the Americas. The answer to this question was “no” given the knowledge at the time. However, the answer could have probably been “yes” (for the Americas) if more attention and research was spent on ZIKV after the initial outbreaks in the Pacific Ocean in 2013 [151]. Although the 2015–2016 Zika epidemic increased awareness, surveillance, diagnostic capacity and in-depth laboratory investigations, ideally preventive surveillance should be expanded to include the well-known pathogens, such as YFV, DENV, CHIKV, ZIKV, RRV and WNV, as well as less recognized ones, such as LACV, MAYV, SLEV, and VEEV. Previously neglected arboviruses such as Oropouche virus, MAYV, and MADV have been in the headlines recently, attracting more attention but funding appears to be a limiting factor. This recognition is likely due to the recent emergence of ZIKV, as well as ongoing globalization driving the geographic expansion of mosquito vectors. However, this list also highlights arboviruses that, as of yet, have not been observed in the Caribbean, nor in the Indian or Pacific Oceans. These pathogens include O’nyong nyong virus, Spondweni virus, Usutu virus, Rocio, Cacicapore virus, Aroa, Naranjal, Bussuquara, and Iguape viruses. These pathogens may not been circulating in these regions, or maybe not detected due to a lack of targeted surveillance in hosts or vectors. Having a clear epidemiological picture in islands is crucial in order to prevent and manage future epidemic crises. There is also a need to expand surveillance and reporting to countries that are connected to islands hotspots through political ties, or high volume of tourism or commerce. Sentinel countries where surveillance should be enhanced are Madagascar and East Africa for the Indian Ocean, and Malaysia and Australia for the Pacific region. For example, in Australia at least 75 arboviruses have been identified, of which several are associated with disease in humans (such Alfuy, Edge Hill, Gan Gan, Kokobera, Sindbis and Stratford viruses), yet not all of them are routinely tested [152]. For the Caribbean region, special attention should be focused on the Brazilian Amazon, where a total of 187 different species of arboviruses were identified between 1954–1998 by the enormous efforts of the Evandro Chagas Institute, and for which very little is known yet [153].

### 4.2. From Challenges to Solutions

In this review, we have focused on arboviral emergence events throughout the tropical island regions. However, reports found in the literature likely underestimate the actual number of emergence events and may not necessarily coincide in time with emergence. In many cases viruses may have been under-reported or under-detected, particularly in developing countries that may not have effective surveillance programs and the capacity to perform viral isolation. We have also included an overview of the factors that render islands vulnerable to epidemics and turn them into amplifying hotspots for connected territories and associated mainland regions. Historical evidence suggests that arboviral diseases have been circulating in the Indian Ocean, Pacific and Caribbean regions for centuries. However, the rapid and explosive pace at which DENV, CHIKV and ZIKV spread throughout these islands in recent times is alarming and demands closer investigation and action. Certainly, the ever-increasing human movement through air-, cruise- and container ship traffic has been shown to be one of the key factors in arboviral spread, particularly across the Caribbean. The recent arboviral epidemics are forcing us to improve our disease prevention and management paradigms.

The epidemiology of arboviruses is complex, involving many intrinsic and extrinsic variables, and for this reason they emerge and re-emerge rather unpredictably. Improving prediction skills would allow improved mitigation strategies. We have summarized below suggestions, which could be implemented to optimize existing efforts:

- Increase efforts to make viral sequence data publically available;
- Enhance surveillance by routinely screening for endemic and exotic viruses (e.g., using genus-level screening tools);
- Increase availability of arboviral surveillance data to connect international public health efforts using platforms such as ProMED;
- Develop real-time tracking (www.nextstrain.org) and forecasting using syndromic or internet search surveillance [154,155];
- Expand the pathogenesis and transmission studies of neglected viruses;
- Search for new viruses using metagenomic and metatranscriptomic approaches that allow for identification and quantification of viral diversity and abundance [156], in addition to classic virology approaches (in vitro cell culture cytopathic effect studies) [157];
- Increase viral banking of outbreak strains to promote evolutionary and phylodynamics studies;
- Expand studies on viral cross-reactivity and immunology;
- Increase field investigations of vectors and potential vertebrate hosts (wildlife and livestock) [158];
- Study vector competence and adaptability [68,159].

Implementation of these items requires a concerted international effort to allocate the necessary resources and coordinate policy. Expanding existing endeavors such as the World Health Organization SIDS Partnership may provide a way forward. This partnership provides a long-term vision and action plan towards sustainable development on island states [162]. The Maldives are an example of a SIDS that is actively engaging in mosquito source reduction as well as disease management strategies including a dengue hotline to connect clinicians across this expansive archipelago with up-to-date advice [163]. An example of a community-based approach is exemplified by the PAHO, the Caribbean Public Health Agency (CARPHA) and the Caribbean Community’s (CARICOM) “Caribbean Mosquito Awareness Week”, an annual event to raise awareness about the connection between mosquitoes and the diseases they transmit and to work with the community to prevent mosquito breeding [164].

Such efforts also require the adoption of a holistic framework, such as provided by the One Health approach, which emphasizes the need for multi-stakeholder collaborations (including, but not limited to, virologists, entomologists, physicians, ecologists, veterinarians, epidemiologists, anthropologists, bio-informaticians, and mathematical modelers). The One Health One Caribbean One Love Project represents an intriguing example [160]. These efforts could be complemented powerfully by web-based surveillance systems [161]. Using geopolitical connectivity data (i.e., maritime commerce routes, tourism), along with analytical tools such as network analysis, it may be possible to determine the probabilities of pathogen spread in a given region. In conclusion, here we highlighted that islands are at high risk for mosquito-borne viral diseases, and this risk is likely to increase. We believe that working towards an interdisciplinary approach and dovetailing into the objectives of existing scientific collaboration and sustainable development efforts is essential to develop a new way forward. Existing trans-national and regional efforts should be supported and expanded to provide novel comprehensive solutions to emerging and re-emerging health issues linked to vector-borne pathogens.

## Figures and Tables

**Table 1:** Emergence and distribution of the mosquito-borne viruses in the islands of the Indian Ocean, Pacific Ocean, and Caribbean area.

Area	Archipelago	Island	Date	Virus	Family	Genus	Origin	Ref.	Comments/ ProMED ref.
Indian Ocean	Seychelles	Seychelles	1977–1978	DENV-2	Flaviviridae	Flavivirus	Human	[25,165]	Genbank: L10048
			1979	CHIKV	Togaviridae	Alphavirus	Human	[38]	Neutralizing antibodies, first report in the Indian Ocean
			1979	SINV	Togaviridae	Alphavirus	Human	[38]	Neutralizing antibodies, first report in the Indian Ocean
			2016	DENV-1	Flaviviridae	Flavivirus	Human	[28]	Recent introduction from China
	Comoros	Grande Comore	1948	DENV	Flaviviridae	Flavivirus	Human	[166]	Probably DENV-1
			1984	DENV-1	Flaviviridae	Flavivirus	Human	[166]	
			1993	DENV-1	Flaviviridae	Flavivirus	Human	[166]	
			2005-2006	CHIKV	Togaviridae	Alphavirus	Human	[167]	
			2007	RFV	Phenuiviridae	Phlebovirus	Human	[37]	First case in Indian Ocean Islands
		Mayotte	1943	DENV	Flaviviridae	Flavivirus	Human	[24]	Unknown serotype
			2004	RVF	Phenuiviridae	Phlebovirus	Livestock	[36]	Sporadic cases in livestock
			1993	DENV-1	Flaviviridae	Flavivirus	Human	[168]	Probably DENV-1
			2005–2006	DENV	Flaviviridae	Flavivirus	Human	[169]	Unknown serotype
			2005–2006	CHIKV	Togaviridae	Alphavirus	Human	[170]	
			2007-2008	RFV	Phenuiviridae	Phlebovirus	Human	[36]	First autochthonous case
			2010	DENV-3	Flaviviridae	Flavivirus	Human	[23,171]	
	Islands of Mascarene	La Réunion	1978	DENV-2	Flaviviridae	Flavivirus	Human	[25,26]	
			2004	DENV-1	Flaviviridae	Flavivirus	Human	[27]	
			2005	CHIKV	Togaviridae	Alphavirus	Human	[39,40]	Introduced from Lamu 2004
			2010	DENV-3	Flaviviridae	Flavivirus	Human	[171]	
		Mauritius	1978–1979	DENV-2	Flaviviridae	Flavivirus	Human	[29,30]	
			2005	CHIKV	Togaviridae	Alphavirus	Human	[172]	
	Maldives Islands	Island not specified	1979	DENV	Flaviviridae	Flavivirus	Human	[173]	Unknown serotype
		Island not specified	2004–2005	DENV	Flaviviridae	Flavivirus	Human	[31,174]	Unknown serotype
		Malé	2006	CHIKV	Togaviridae	Alphavirus	Human	[175]	Malé and other islands of the Maldives
		Malé	2011	DENV-1	Flaviviridae	Flavivirus	Human	[32,173]	Largest epidemic, Malé and other islands of the Maldives
		Dhiffushi	2015	ZIKV	Flaviviridae	Flavivirus	Human	[176]	
	Andaman and Nicobar Islands	Island not specified	1988	CHIKV	Togaviridae	Alphavirus	Human	[33]	Serological survey
		Island not specified	1988	DENV-2	Flaviviridae	Flavivirus	Human	[33]	Serological survey
		Island not specified	1988	JEV	Flaviviridae	Flavivirus	Human	[33]	Serological survey
		Island not specified	1988	WNV	Flaviviridae	Flavivirus	Human	[33]	Serological survey
		Island not specified	1988	KFD	Flaviviridae	Flavivirus	Human	[33]	Serological survey
		Island not specified	1988	LGTV	Flaviviridae	Flavivirus	Human	[33]	Serological survey
		Island not specified	2009–2010	DENV-1	Flaviviridae	Flavivirus	Human	[34]	
		Island not specified	2013	DENV-3	Flaviviridae	Flavivirus	Human	[35]	
Pacific	Polynesia	American Samoa	1972	DENV-2	Flaviviridae	Flavivirus	Human	[52]	
			1975	DENV-1	Flaviviridae	Flavivirus	Human	[52]	
			1979	RRV	Togaviridae	Alphavirus	Human, rat, dog, pig, chicken	[177]	
			2014	CHIKV	Togaviridae	Alphavirus	Huaan	[20,41]	
			2016	ZIKV	Flaviviridae	Flavivirus	Human	[178]	
		Cook Islands	1980	RRV	Togaviridae	Alphavirus	Human, pig, dogs, cattle, mosquitoes	[179]	
			2014	ZIKV	Flaviviridae	Flavivirus	Human	[151] ProMED	http://www.promedmail.org/post/20140328.2365267
		French Polynesia	1967	DENV-3	Flaviviridae	Flavivirus	Human	[180]	Tahiti
			1971	DENV-2	Flaviviridae	Flavivirus	Human	[181]	Tahiti
			1975	DENV-1	Flaviviridae	Flavivirus	Human	[182]	
			1979	DENV-4	Flaviviridae	Flavivirus	Human	[182]	
			2013	ZIKV	Flaviviridae	Flavivirus	Human	[151,183]	
			2014	CHIKV	Togaviridae	Alphavirus	Human	[20,41]	CHIKV imported into French Polynesia from a traveller returning from Guadeloupe
		Hawaii	1912	DENV	Flaviviridae	Flavivirus	Human	[184]	Undetermined serotype
			2001	DENV-1	Flaviviridae	Flavivirus	Human	[43]	Traveler returning from French Polynesia
			2004–2005	JEV, SLEV	Flaviviridae	Flavivirus	Resident and migratory birds	[185]	
			2015	DENV	Flaviviridae	Flavivirus	Human	[186,187]	Undetermined serotype
		Samoa	2014	ZIKV	Flaviviridae	Flavivirus	Human	[183]	
			2014	CHIKV	Togaviridae	Alphavirus	Human	[41]	
		Tokelau	2014	ZIKV	Flaviviridae	Flavivirus	Human	[183]	
			2014	CHIKV	Togaviridae	Alphavirus	Human	[41]	
		Tonga	1974	DENV-2	Flaviviridae	Flavivirus	Human	[188]	
			1975	DENV-1	Flaviviridae	Flavivirus	Human	[182]	
			2014	CHIKV	Togaviridae	Alphavirus	Human	[41]	
			2014	ZIKV	Flaviviridae	Flavivirus	Human	[183]	
		Tuvalu	1974	DENV-1	Flaviviridae	Flavivirus	Human	[182]	
		Wallis and Fatuna Island	1975	RRV	Togaviridae	Alphavirus	Human	[189]	
			1976	DENV-1 DENV-4	Flaviviridae	Flavivirus	Mosquitoes	[190]	
		Easter Island	2014	ZIKV	Flaviviridae	Flavivirus	Human	[191]	
		Niue	1972	DENV-2	Flavivirida	Flavivirus	Human	[192]	DHF
	Micronesia	Marshall Islands	1974	DENV-1	Flaviviridae	Flavivirus	Human	[182]	
			2016	ZIKV	Flaviviridae	Flavivirus	Human	[193]	
		Nauru	1974	DENV-1	Flaviviridae	Flavivirus	Human	[182]	
		Palau	1995	DENV-4	Flaviviridae	Flavivirus	Human	[194]	
			2000	DENV-1	Flaviviridae	Flavivirus	Human	[42]	
			2016	ZIKV	Flaviviridae	Flavivirus	Human	[195]	Locally acquired
		Kiribati	1974	DENV-1	Flaviviridae	Flavivirus	Human	[182]	
			2016	ZIKV	Flaviviridae	Flavivirus	Human	[195]	
		Yap Island	1995	DENV-4	Flaviviridae	Flavivirus	Human	[196]	
			2004	DENV-1	Flaviviridae	Flavivirus	Human	[197]	
			2007	ZIKV	Flaviviridae	Flavivirus	Human	[15]	
			2013	CHIKV	Togaviridae	Alphavirus	Human	[198] ProMED	http://www.promedmail.org/post/20140114.2171369
	Melanesia	Fiji	1943–1944	DENV	Flaviviridae	Flavivirus	Human	[199]	First report but unknown serotype
			1959–1960	DENV-2	Flaviviridae	Flavivirus	Human	[199]	
			1959–1960	DENV-1	Flaviviridae	Flavivirus	Human	[199]	
			1959–1960	JEV	Flaviviridae	Flavivirus	Human, fowls	[199]	
			1979	RRV	Togaviridae	Alphavirus	Human	[46] ProMED	http://www.promedmail.org/post/20140328.2365267
			2015	ZIKV	Flaviviridae	Flavivirus	Human	[193]	
		New Caledonia	1971	DENV-2	Flaviviridae	Flavivirus	Human	[200]	
			1975	RRV	Togaviridae	Alphavirus	Mosquitoes	[189]	
			1975	DENV-1 DENV-4	Flaviviridae	Flavivirus	Human	[201]	
			1979–1980	RRV	Togaviridae	Alphavirus	Human	[202]	
			1989	DENV-3	Flaviviridae	Flavivirus	Human	[203]	
			2011	CHIKV	Togaviridae	Alphavirus	Human	[204]	GenBank: HE806461
			2013	ZIKV	Flaviviridae	Flavivirus	Human	[151]	Imported French Polynesia
		Vanuatu	1975	DENV-1 DENV-4	Flaviviridae	Flavivirus	Mosquitoes	[182]	
			1975	RRV	Togaviridae	Alphavirus	Mosquitoes	[189]	
			2015	ZIKV	Flaviviridae	Flavivirus	Human	ProMED	http://www.promedmail.org/post/20150501.3334549
		Solomon Islands	1982	DENV-3	Flaviviridae	Flavivirus	Human	[182]	
			2015	ZIKV	Flaviviridae	Flavivirus	Human	[193]	
			2016	DENV-2	Flaviviridae	Flavivirus	Human	[44]	
		Papua New Guinea	1956–1957	MVE	Flaviviridae	Flavivirus	Human	[205]	Serological survey
			1963	DENV-1	Flaviviridae	Flavivirus	Human	[206]	
			1963	DENV-2	Flaviviridae	Flavivirus	Human	[206]	
			1971	SINV	Togaviridae	Alphavirus	Human	[207]	
			1989	JEV	Flaviviridae	Flavivirus	Human	[208]	
			2012	ZIKV	Flaviviridae	Flavivirus	Human	[20]	
			2012	CHIKV	Togaviridae	Alphavirus	Human	[209]	
			2016	DENV-3	Flaviviridae	Flavivirus	Human	[44]	
Caribbean	Lucayan	Turks and Caicos Islands	2014	CHIKV	Togaviridae	Alphavirus	Human	[140]	
			2016	ZIKV	Flaviviridae	Flavivirus	Human	[210]	
		The Bahamas	2003	WNV	Flaviviridae	Flavivirus	Human	[77]	Second case in the Caribbean
			2014	CHIKV	Togaviridae	Alphavirus	Human	[140]	
			2016	ZIKV	Flaviviridae	Flavivirus	Human	[210]	
	Greater Antilles	Cayman Islands	2001	WNV	Flaviviridae	Flavivirus	Human	[77]	First Case of WNV in the Caribbean (August 2001) http://www.promedmail.org/post/20011016.2538
			2014	CHIKV	Togaviridae	Alphavirus	Human	[140]	
			2016	ZIKV	Flaviviridae	Flavivirus	Human	[210]	
		Cuba	1969	EEEV	Togaviridae	Alphavirus	Field	[62]	
			1981	DENV-2	Flaviviridae	Flavivirus	Human	[211]	This case occurred four years after DENV-1 had been introduced. First case of Dengue hemorrhagic fever (DHF) in the Americas
			2012	DENV-4	Flaviviridae	Flavivirus	Human	[212]	DHF
			2004	WNV	Flaviviridae	Flavivirus	Migratory birds	[77]	
			2005	WNV	Flaviviridae	Flavivirus	Human, horses	[77]	
			2014	CHIKV	Togaviridae	Alphavirus	Human	[140]	
			2016	ZIKV	Flaviviridae	Flavivirus	Human	[210]	
		Dominican Republic	2002	WNV	Flaviviridae	Flavivirus	Horses, chickens and resident birds	[77]	
			2014	CHIKV	Togaviridae	Alphavirus	Human	[213]	
			2015	ZIKV	Flaviviridae	Flavivirus	Human	ProMED	According to PAHO, the first case in the Caribbean emerged in November 2015. This case was reported on May 2015, may be the first Zika case outside of Brazil in the Americas. http://www.promedmail.org/post/20150605.3412519
		Haiti	1969–1971	DENV-2	Flaviviridae	Flavivirus	Human, mosquitoes	[214]	DHF
			1972	WNV, SLEV, EEEV	Flaviviridae, Togaviridae	Flavivirus, Alphavirus	Birds and bats	[215]	
			1994	DENV-1 DENV-4	Flaviviridae	Flavivirus	Human	[216]	
			2013	CHIKV	Togaviridae	Alphavirus	Human	[217]	
			2014–2015	MAYV	Togaviridae	Alphavirus	Human	[66]	
			2016	CHIKV	Flaviviridae	Flavivirus	Mosquitoes	[218]	New ECSA subgroup
			2016	ZIKV	Flaviviridae	Flavivirus	Human	[219]	
			2016	SPONV	Flaviviridae	Flavivirus	Mosquito	[220]	
		Jamaica	1956	DENV-2	Flaviviridae	Flavivirus	Human	[221]	
			1963	DENV-3	Flaviviridae	Flavivirus	Human	[59]	
			1962	EEEV	Togaviridae	Alphavirus	Human	[221]	
			1966	CVV	Peribunyaviridae	Orthobunyavirus	Mosquitoes	[222]	
			1960–1975	SLEV	Flaviviridae	Flavivirus	Human	[221]	
			1977	DENV-1	Flaviviridae	Flavivirus	Human	[60]	First detection in the Americas, probably from Africa
			2002	WNV	Flaviviridae	Flavivirus	Birds	[77]	
			2014	CHIKV	Togaviridae	Alphavirus	Human	[13,140,223]	
			2016	ZIKV	Flaviviridae	Flavivirus	Human	[224]	
		Puerto Rico	2004	WNV	Flaviviridae	Flavivirus	Migratory birds	[77,225]	
			1963	DENV-3	Flaviviridae	Flavivirus	Human	[59,226]	First virological confirmation of DENV in Puerto Rico
			1969	DENV-2	Flaviviridae	Flavivirus	Human	[226]	
			1977	DENV-1	Flaviviridae	Flavivirus	Human	[226]	Second case in the Caribbean
			1981	DENV-4	Flaviviridae	Flavivirus	Human	[227]	
			2007	WNV	Flaviviridae	Flavivirus	Human	[228]	
			2014	CHIKV	Togaviridae	Alphavirus	Human	[13,140,223]	
			2015	ZIKV	Flaviviridae	Flavivirus	Human	[229]	
	Greater Antilles	US Virgin Islands	1981	DENV-4	Flaviviridae	Flavivirus	Human	[230]	
			2014	CHIKV	Togaviridae	Alphavirus	Human	[13,223] ProMED	http://www.promedmail.org/post/20140614.2539532
			2016	ZIKV	Flaviviridae	Flavivirus	Human	[210]	
		British Virgin Islands	2014	CHIKV	Togaviridae	Alphavirus	Human	[13] ProMED	http://www.promedmail.org/post/20140114.2172714
			2016	ZIKV	Flaviviridae	Flavivirus	Human	[210]	
		Anguilla	1964	DENV-3	Flaviviridae	Flavivirus	Human	[59]	
			2014	CHIKV	Togaviridae	Alphavirus	Human	[13,140,223]	
			2016	ZIKV	Flaviviridae	Flavivirus	Human	[210]	
		Antigua and Barbuda	1964	DENV-3	Flaviviridae	Flavivirus	Human	[59]	
			2014	CHIKV	Togaviridae	Alphavirus	Human	ProMED	http://www.promedmail.org/post/20140427.243388
			2016	ZIKV	Flaviviridae	Flavivirus	Human	[210]	
		Saint Martin	2010–2012	DENV-1 DENV-2 DENV-4	Flaviviridae	Flavivirus	Human	[231]	
			2013	CHIKV	Togaviridae	Alphavirus	Human	ProMED	First local transmission in the Western Hemisphere http://www.promedmail.org/post/20131209.2099940
			2016	ZIKV	Flaviviridae	Flavivirus	Human	[210]	
		Saba	2016	ZIKV	Flaviviridae	Flavivirus	Human	[210]	
		St Eustatius	2014	CHIKV	Togaviridae	Alphavirus	Human	[13,223] ProMED	http://www.promedmail.org/post/20140922.2794035
			2016	ZIKV	Flaviviridae	Flavivirus	Human	[210]	
		Saint Barthélemy	1981	DENV-4	Flaviviridae	Flavivirus	Human	[230]	
			2010–2012	DENV-1 DENV-2	Flaviviridae	Flavivirus	Human	[231]	
			2014	CHIKV	Togaviridae	Alphavirus	Human	[13,223] ProMED	http://www.promedmail.org/post/20140102.2148245
			2016	ZIKV	Flaviviridae	Flavivirus	Human	[210]	
		Saint Kitts and Nevis	1964	DENV-3	Flaviviridae	Flavivirus	Human	[59]	
			2014	CHIKV	Togaviridae	Alphavirus	Human	[13,223] ProMED	http://www.promedmail.org/post/20140220.229088
			2016	ZIKV	Flaviviridae	Flavivirus	Human	[210]	
		Montserrat	2014	CHIKV	Togaviridae	Alphavirus	Human	[223]	
			2016	ZIKV	Flaviviridae	Flavivirus	Human	[210]	
		Guadeloupe	2002	WNV	Flaviviridae	Flavivirus	Horses, chickens, resident birds	[77]	
			2013	CHIKV	Togaviridae	Alphavirus	Human	[13,223] ProMED	http://www.promedmail.org/post/20131227.2139145
			2016	ZIKV	Flaviviridae	Flavivirus	Human	[210]	
	Windward Islands	Dominica	1981	DENV-4	Flaviviridae	Flavivirus	Human	[232]	First detection in the Americas, probably introduced from Pacific Islands
			2014	CHIKV	Togaviridae	Alphavirus	Human	[13,223] ProMED	http://www.promedmail.org/post/20140118.2181292
			2016	ZIKV	Flaviviridae	Flavivirus	Human	[210]	
		Martinique	1964	DENV-3	Flaviviridae	Flavivirus	Human	[59]	
			2013	CHIKV	Togaviridae	Alphavirus	Human	[13,223] ProMED	http://www.promedmail.org/post/20131209.209994
			2015	ZIKV	Flaviviridae	Flavivirus	Human	[210]	
		Saint Lucia	2014	CHIKV	Togaviridae	Alphavirus	Human	[140]	
			2016	ZIKV	Flaviviridae	Flavivirus	Human	[210]	
		Saint Vincent and the Gredines	2014	CHIKV	Togaviridae	Alphavirus	Human	[140]	
			2016	ZIKV	Flaviviridae	Flavivirus	Human	[210]	
		Grenada	2014	CHIKV	Togaviridae	Alphavirus	Human	[233]	
			2016	ZIKV	Flaviviridae	Flavivirus	Human	[210]	
		Barbados	2014	CHIKV	Togaviridae	Alphavirus	Human	ProMED	http://www.promedmail.org/post/20140809.2674777
			2016	ZIKV	Flaviviridae	Flavivirus	Human	[210]	
		Trinidad and Tobago	1953	DENV-2	Flaviviridae	Flavivirus	Human	[56]	First introduction to the Americas
			1954	MAYV	Togaviridae	Alphavirus	Human	[65]	
			1955	OROV	Peribunyaviridae	Orthobunyavirus	Mosquitoes	[69]	
			1955–1959	BSBV, IERIV, LUKV	Peribunyaviridae	Orthobunyavirus	Mosquitoes	[70]	
			1957	SLEV	Flaviviridae	Flavivirus	Birds and mosquitoes	[71,72]	
			1962	ILHV	Flaviviridae	Flavivirus	Human	[74]	
			1965	RESV	Peribunyaviridae	Orthobunyavirus	Mosquitoes	[75]	
			2004	WNV	Flaviviridae	Flavivirus	Horses and birds	[77]	
			2006-2009	MADV	Togaviridae	Alphavirus	horses	[73]	
			2007	MUCV	Togaviridae	Alphavirus	Mosquitoes, mammals	[64]	
			2014	CHIKV	Togaviridae	Alphavirus	Human	[140]	
			2016	ZIKV	Flaviviridae	Flavivirus	Human	[210]	
	Leeward Antilles	Aruba	2014	CHIKV	Togaviridae	Alphavirus	Human	[13] ProMED	http://www.promedmail.org/post/20140205.2257138
			2016	ZIKV	Flaviviridae	Flavivirus	Human	[210]	
		Curaçao	2014	CHIKV	Togaviridae	Alphavirus	Human	[13] ProMED	http://www.promedmail.org/post/20140809.2674777
			2016	ZIKV	Flaviviridae	Flavivirus	Human	[210]	
		Bonaire	2016	ZIKV	Flaviviridae	Flavivirus	Human	[210]	

Dengue virus (DENV), Chikungunya virus (CHIKV), Rift Valley fever (RVF), Zika virus (ZIKV), Japanese encephalitis (JEV), West Nile virus (WNV), Kyasanur Forest disease (KFD), Langat virus (LGTV), Ross River virus (RRV), Murray Valley encephalitis (MVE), Eastern equine encephalomyelitis virus (EEEV), Saint Louis encephalitis (SLEV), Mayaro virus (MAYV), Oropuche virus (OROV) Cache Valley virus (CVV), Bushbush virus (BSBV), Ieri virus (IERIV), Lukuni virus (LUKV), Ilheus virus (ILHV), Restan virus (RESV), Madariaga virus (MADV), Mucambo virus (MUCV), World Health Organization (WHO).

## Supplementary Materials

The following are available online at http://www.mdpi.com/1999-4915/11/1/11/s1, Table S1: Table S1. Arboviral imported cases in Florida (2002–2018). Table S2: Florida Arboviral imported cases from the Caribbean (2012–2018).
