# Supplementary material for: Islands as Hotspots for Emerging Mosquito-Borne Viruses: A One-Health Perspective
Source: Viruses. 2018 Dec 25;11(1):11. doi: 10.3390/v11010011 (PMC6356932; doi:10.3390/v11010011)
Supplement: Supplementary file 1 [file viruses-11-00011-s001.pdf]

Supplementary

Islands As Hotspots for Emerging Mosquito-Borne Viruses: A One-Health Perspective

Table S1. Arboviral imported cases in Florida (2002–2018).

Year	WNV	SLEV	EEEV	LACV	DENV	CHIKV	ZIKV
**2002**	35 local	1	1	0	12 (imported)	0	0
**2003**	94 local	0	3	0	0	0	0
**2004**	42 local	0	0	0	0	0	0
**2005**	21 local	0	5	0	18 (imported)	0	0
**2006**	3 (imported)	0	0	0	21 (imported)	0	0
**2007**	3 (2 local, 1 imported)	0	0	1 (imported)	53 imported (19 Puerto Rico, 12 Dominican Republic, 22 Latin America, Asia, and Africa)	0	0
**2008**	3 (2 local, 1 imported)	0	1	1 (imported)	25 imported (5 Puerto Rico, 5 Dominican Republic, 1 Haiti, 1 Jamaica, 1 St. Thomas, 1 Jamaica, 1 Caribbean, 1 Brazil, 1 Carcao, 1 Costa Rica, 1 Honduras, 1 Malaysia, 1 Thailand, 1 Venezuela, and 1 Asia/Africa, 1 unknown)	0	0
**2009**	3 (2 local, 1 imported)	0	0	0	22 imported (3 Dominican Republic, 3 Puerto Rico, 10 Haiti, 2 Honduras, 2 Guatemala, 2 Bolivia, Brazil, 2 Colombia, 3 1 India, 1 Malaysia, 1 Mexico, 1 Nicaragua, 3 Panama, 1 Philippines, 1 Suriname)	0	0
**2010**	12 local	0	4	0	198 (65 local, 133 imported 5 Jamaica, 2 Martinique, 1 Maldives, 1 Cayman Islands, 1	0	0

					Cuba, 13 Dominican Republic, 6 Haiti, 36 Puerto Rico, 1 Trinidad, 3 Virgin Islands, 1 Bangladesh, 1 Brazil, 8 Colombia, 4 Costa Rica, 1 Ecuador, 1 El Salvador, 1 Ghana, 4 Grenada, 2 Guatemala, 6 Honduras, 1 Mexico, 13 Nicaragua, 1 Pakistan, 1 Philippines, 1 Thailand, 16 Venezuela, 1 Malaysia/Dubai/Bangladesh, 1 Panama/Venezuela)		
**2011**	24 local	0	0	0	68 (7 local, 61 imported 1 Aruba, 14 Bahamas, 11 Puerto Rico, 2 St. Lucia, 4 Trinidad, 1 Turks and Caicos Islands, 5 Cuba, 1 Dominican Republic, 2 Jamaica, 2 Haiti, 3 Bangladesh, 3 Brazil, 1 Colombia, 1 Costa Rica, 1 Grenada, 1 Guyana, 1 India, 2 Nicaragua, 1 Pakistan, 2 Panama, 1 Venezuela, 1 Vietnam)	0	0
**2012**	68 (62 local, 6 imported)	0	1	0	139 (4 local, 135 imported 1 Brazil, 1 Colombia, 29 Cuba, 17 Haiti, 17 Dominican Republic, 4 Trinidad, 1 Turks & Caicos, 1 Virgin Islands, 1 St. Vincent, 23 Jamaica, 16 Puerto Rico, 4 Philippines, 4 Ecuador, 2 El Salvador, 1 Ghana, 2 Guyana, 1 Honduras, 1 India, 2 Mexico, 2 Nicaragua, 1 Panama, 1 Portugal, 1 South Africa, 1 Sri Lanka, 1 Suriname)	0	0

**2013**	5 local	0	2	0	143 (23 local, 120 imported 1 U.S. Virgin Islands, 46 Puerto Rico, 2 Saint Martin, 1 Trinidad, 6 Cuba, 7 Haiti, 1 Dominica, 12 Dominican Republic, 1 Caribbean, 1 Barbados, 2 Philippines, 1 Angola, 2 Bangladesh,1 Bolivia, 3 Brazil, 5 Columbia, 4 Costa Rica, 4 Honduras, 1 India, 1 Indonesia, 5 Jamaica, 2 Mexico, 3 Nicaragua, 2 Nigeria, 2 Panama, 4 Venezuela)	0	0
**2014**	21 (20 local: 4 blood donor, 1 imported Bahamas)	2	0	1 (imported)	86 (6 local, 80 imported 1 Trinidad, 1 Bangladesh, 1 Bolivia, 2 Brazil, 1 Caribbean, 1 Columbia, 5 Costa Rica, 27 Cuba, 1 Cuba/Bahamas, 9 Dominican Republic, 2 El Salvador, 1 Guadeloupe, 1 Guatemala, 1 Guyana, 4 Haiti, 7 Honduras, 2 Jamaica, 3 Mexico, 6 Puerto Rico, 1 Sri Lanka, 3 Venezuela)	562 (11 local, 552 imported 2 Antigua, 1 Antigua/Barbuda, 1 Barbados, 1 Bequia, 1 Caribbean, 4 Colombia, 7 Cuba, 1 Dominica/Guadalupe, 88 Dominican Republic, 6 El Salvador, 1 Grenada, 1 Guatemala, 10 Guyana, 107 Haiti, 1 Haiti/Dominican 1 Republic, 2 Honduras, 1 India, 1 Indonesia, 66 Jamaica, 2 Martinique, 3 Nicaragua, 1 Philippines, 119 Puerto Rico, 2 Puerto Rico/Dominican Republic, 1 South America, 2 St. Lucia, 2	0

						St. Thomas/St. Martin/Bahamas, 1 St. Vincent and the Grenadines, 6 Trinidad, 3 Venezuela, 8 Virgin Islands)	
**2015**	11 local (2 blood donor)	0	0	1 (imported)	83 (1 local, 82 imported 1 Jamaica, 26 Cuba, 6 Dominican Republic, 1 Puerto Rico, 11 Haiti, 3 Hawaii, 5 Brazil, 1 Colombia, 3 Costa Rica, 1 El Salvador, 1 Guatemala, 1 Honduras, 1 Bangladesh, 3 India, 3 Mexico, 2 Nicaragua, 4 Philippines, 1 Thailand, 7 Venezuela, 1 Vietnam)	73 (imported 1 Bolivia, 13 Colombia, 1 Dominican Republic, 1 Ecuador, 3 El Salvador, 4 Guatemala, 2 Haiti, 6 Honduras, 2 India, 3 Jamaica, 11 Mexico, 19 Nicaragua, 4 Puerto Rico, 1 Trinidad, 1 Venezuela, 1 Virgin Islands)	0
**2016**	6 local (1 blood donor)	0	2 (1 imported)	1 (imported)	45 (2 local, 43 imported 2 Puerto Rico, 1 St. Barthelemy, 3 Haiti, 7 Cuba, 2 Dominican Republic, 6 Jamaica, 3 Brazil, 3 Colombia, 5 Costa Rica, 1 El Salvador, 1 Ghana, 1 Honduras, 2 India, 1 Indonesia, 1 Mexico, 1 Singapore, 3 Venezuela)	6 (imported 1 Bahamas/Mexico, 1 Jamaica, 2 Brazil, 1 Bolivia)	1272 (49 undetermined, 256 local, 1016 imported: 1 Anguilla, 3 Antigua and Barbuda, 1 Aruba/Curacao, 9 Bahamas, 2 Bahamas/Virgin Islands, 7 Barbados, 2 Barbados/Dominica, 22 Virgin Islands, 28 Trinidad, 1 Brazil/Caribbean, 1 Brazil/Haiti, 4 Caribbean, 1 Cayman Islands, 1 Dominica, 139 Dominican Republic, 3 Dominican Republic/Puerto Rico, 1 Dominican Republic/Venezuela, 11 Cuba, 80 Haiti, 1 Haiti/Virgin Islands, 1 Mexico/Trinidad, 1 Martinique, 212 Puerto Rico, 2 St. Barthelemy/St. Martin, 3 St. Eustatius, 1 St. Lucia, 3 St. Martin, 1 Mexico/Caribbean, 2 Belize, 4 Bolivia, 8 Brazil, 1 Brazil/Bolivia/Peru, 1 Central America, 40 Colombia, 4 Costa Rica, 1 Costa Rica/Nicaragua, 2 Curacao, 1 Curacao/Guyana, 2 Ecuador, 7 El Salvador, 4 Grenada, 3 Guadeloupe, 15 Guatemala, 6 Guyana, 36 Honduras, 1 Honduras/Guatemala, 1 Honduras/Mexico,

							109 Jamaica, 3 Jamaica/Panama, 31 Mexico, 1 Mexico/Panama, 117 Nicaragua, Panama, 1 Suriname, 70 Venezuela)
**2017**	4 local (2 blood donor)	0	1	0	18 imported (2 Jamaica, 6 Cuba, 1 Cuba/Mexico, 1 Guatemala, 3 India, 1 Nigeria, 1 Bhutan/India, 1 Brazil, 1 Pakistan, 1 Philippine)	4 imported (1 Brazil, 3 India)	289 (35 undetermined, 3 sexual transmission, 2 local, 207 imported: 2 Barbados, 6 Puerto Rico, 79 Cuba, 1 Saint Kitts and Nevis, 1 Barbados/Virgin Islands, 39 Haiti, 1 Haiti/Dominican Republic, 10 Dominican Republic, 1 Dominican Republic/Puerto Rico, 8 Jamaica, 1 Trinidad, 1 Cayman Islands/St. Martin, 1 Philippines, 1 Bolivia, 2 Colombia, 1 Costa Rica, 3 Curacao, 3 Ecuador, 2 El Salvador, 2 Guatemala, 6 Honduras, 5 Mexico, 1 Mexico/Thailand, 1 Nicaragua, 1 Panama, 1 Sri Lanka, 17 Venezuela, 7 multiple countries)
**2018**	1 local (blood donor)	0	1	0	10 imported (5 Cuba, 4 Haiti, 1 Venezuela)	1 imported (Brazil)	59 imported (13 Cuba, 1 Cuba/Mexico, 1 Dominica, 1 Dominican Republic, 1 Jamaica, 1 Puerto Rico, 20 Haiti, 1 Haiti/Brzil, 3 Brazil, 1 Brazil/Mexico, 2 Guatemala, Guatemala/Mexico, 4 Honduras, 9 Venezuela)

West Nile virus (WNV), Eastern equine encephalomyelitis virus (EEEV), Saint Louis encephalitis (SLEV), LaCrosse encephalitis (LACV), Dengue virus (DENV), Chikungunya virus (CHIKV), Zika virus (ZIKV). Data obtained from FDoH (http://www.floridahealth.gov/diseases-and-conditions/mosquito-borne-diseases/surveillance.html).

Table S2. Florida Arboviral imported cases from the Caribbean (2012–2018).

Year	Arbovirus	Imported cases	Puerto Rico	Dominican Republic	Haiti	Cuba	Jamaica	Other Caribbean Island	% Imported cases Caribbean
2008	DENV	25	5	5	1		1	2	56.0
2009	DENV	22	3	3	10		1		77.3
2010	DENV	133	36	13	6	1	5	7	51.1
2011	DENV	61	11	1	2	5	2	22	70.5
2012	DENV	135	16	17	17	29	23	7	80.7
2013	DENV	120	46	12	7	6		7	65.0
2014	DENV	80	6	9	4	27	2	4	65.0
	CHIKV	552	119	90	108	7	66	25	75.2
2015	DENV	83	1	6	11	26	1		54.2
	CHIKV	73	4	1	2		3	2	16.4
2016	DENV	43	2	2	3	7	6	1	48.8
	CHIKV	6					1	1	33.3
	ZIKV	1016	215	140	82	11		97	53.6
2017	DENV	18				7	2		50.0
	ZIKV	207	6	12	39	79	8	6	72.5
2018	DENV	10			4	5			90.0
	ZIKV	59	1	1	21	14	1	1	66.1
TOT Imported cases 2008-2018		2643	471	312	317	224	122	182	
% Caribbean cases 2008-2018		61.6							

Dengue virus (DENV), Chikungunya virus (CHIKV), Zika virus (ZIKV).
